# Cross-cultural adaptation and validation of hospital survey on patient safety culture in Bengali version (B-HSOPSC 2.0) among nurses in Bangladesh: a cross-sectional study

**DOI:** 10.1186/s12912-026-04817-3

**Published:** 2026-05-29

**Authors:** Md Abdul Khalek, K. A. T. M. Ehsanul Huq, Tomonori Hasegawa, Yosuke Hatakeyama, Abdulfatai Olamilekan Babaita, Michiko Moriyama

**Affiliations:** 1 Graduate School of Biomedical and Health Sciences, Hiroshima University, Hiroshima, Japan; 2 Department of Social Medicine, Faculty of Medicine, Toho University, Tokyo, Japan

**Keywords:** Cross-cultural, Safety culture, Hospital survey, Patient safety, Reliability

## Abstract

**Background:**

The Hospital Survey on Patient Safety Culture (HSOPSC 2.0) was developed and updated by the Agency for Healthcare Research and Quality in 2019. It has now been widely adopted and translated into different languages worldwide. However, the validity and reliability of the Bengali version of HSOPSC 2.0 (B-HSOPSC 2.0) have not been tested among healthcare professionals. This study aimed to determine the validity and reliability of the B-HSOPSC 2.0 with cross-cultural adaptations, among hospital nurses in Bangladesh.

**Methods:**

The study was conducted among nurses in eight tertiary-level government medical college hospitals in Bangladesh. A two-step study design was employed, encompassing the translation, cultural adaptation, and psychometric evaluation of B-HSOPSC 2.0. The translation process included forward and backward translation by panel, expert consensus, review, and pretesting. Content validity, reliability, and test-retest reliability were assessed. Construct validity was evaluated through confirmatory factor analysis (CFA), and convergent validity was examined using average variance extraction and Spearman’s correlation coefficient.

**Results:**

Out of 7,170 eligible nurses, 4,982 were analyzed (valid response rate: 69.5%), and a subset (*n* = 424) provided retest responses. The words “manager” and “clinical leader” were removed, and the word “units” was replaced with “wards,” as they were deemed inappropriate for the Bangladesh healthcare system. The content validity index provides strong evidence of adequacy for the instrument measurements (I-CVI = 0.83-1.00, S-CVI = 0.98). B-HSOPSC 2.0 demonstrated a good internal consistency (Cronbach’s α = 0.70–0.76). The test-retest reliability was acceptable at the group level (ICC = 0.65–0.76) and low in the single measure (0.071, 0.069). In the CFA model, the indices for the 10 and 9 dimensions were CFI = 0.79, 0.83; NFI = 0.78, 0.82; TLI = 0.74, 0.79; GFI = 0.91, 0.92; RMSEA = 0.06, 0.06; SRMR = 0.07, 0.06, respectively.

**Conclusions:**

The psychometric properties of the Bengali version were inconsistent and not sufficiently robust. However, content validity and test-retest reliability were deemed acceptable. Since there is no validated tool to evaluate the patient safety culture in the Bengali version, these study findings may help further to evaluate future nurses’ perceived patient safety culture interventions in hospital settings in Bangladesh. Further studies are required, as the psychometric properties of B-HSOPSC 2.0 among other healthcare professionals in Bangladesh remain to be confirmed.

**Clinical trial number:**

Not applicable.

**Supplementary Information:**

The online version contains supplementary material available at 10.1186/s12912-026-04817-3.

## Introduction

Patient safety is a fundamental pillar of high-quality healthcare, essential for preventing avoidable harm and ensuring safe healthcare worldwide [1]. Healthcare itself can cause harm that originates largely from unsafe systems and organizational factors, rather than individual errors [2, 3]. The World Health Organization (WHO) convened international experts and policymakers for effective collaboration on patient safety action globally [4]. WHO defined patient safety as protecting patients from preventable harm and minimizing the risk of unnecessary injury during healthcare to the lowest possible level [5]. The National Institute for Occupational Safety and Health (NIOSH) explained that safety culture is an organizational commitment to health and safety, reflected in shared values, attitudes, perceptions, and behaviors of individuals or groups characterized by mutual trust, open communication, and confidence in preventive measures [6]. The culture of patient safety is the sum of beliefs and practices that shape the way healthcare is provided [7].

Lack of a patient safety culture can cause adverse events due to inadequate medical management rather than underlying illness, such as medication errors, falls, infections, pressure ulcers, and equipment failures [8, 9]. It was estimated that 1 in every 10 patients in high-income countries (HICs) experiences adverse events, and 2.6 million people in low-and middle-income countries (LMICs) died due to unsafe care in hospitals in 2019 [10]. In LMICs, unsafe healthcare affects up to 40% of patients, resulting in more than 3 million deaths annually [5]. About half of these incidents are preventable, with many linked to medication errors [10]. Moreover, LMICs confront challenges such as workforce shortages, fragmented care, and inadequate infrastructure, which amplify risks and make patient safety a critical public health concern [11]. It is anticipated that if the patient safety culture is continuously evaluated and improved, patient outcomes and overall safety will be increased [12, 13].

Nurses, the largest group of healthcare providers, play a key role in preventing errors [14], and their proficiency is essential for identifying risks, warning signs, and fostering a culture of patient safety [15]. Preventing safety errors and improving quality of care depend on nurses’ competency and principal of patient safety [16]. A study finding from Bangladesh revealed gaps in infection control knowledge and practices of nurses, emphasizing the need to strengthen patient safety initiatives [17]. Therefore, continuous improvement of nurses’ ability is essential to maintain a successful and comprehensive patient safety strategy [18].

Strengthening patient safety culture is a global priority, with experts recommending validated tools for precise assessment and targeted improvement [19, 20]. Various patient safety survey instruments are used worldwide to assess organizational safety, environment, and culture. Notable instruments include the Surveys on Patient Safety Culture™ (SOPS^®^) by Agency for Healthcare Research and Quality (AHRQ) [7], the Safety Attitudes Questionnaire (SAQ) by University of Texas [21] the Manchester Patient Safety Framework (MaPSaF) by University of Manchester [22] and the Patient Safety Climate in Healthcare Organizations (PSCHO) by Stanford University [23]. The SAQ is shorter and effectively measures perceptions of teamwork and safety climate; however, it offers limited insight into broader organizational culture factors [21]. In contrast, MaPSaF offers a qualitative and multidimensional assessment of safety culture; however, it lacks standardization and is difficult to benchmark quantitatively [24]. The PSCHO used to assess organizational climate also has notable weaknesses, including poor internal consistency, lack of confirmatory factor analysis (CFA), low response rates, and limited generalizability beyond U.S. settings [23]. HSOPSC 2.0 is a standardized widely used tool that provides benchmarking data and identifies improvement areas, while SAQ helps to address only concerns, foster a positive culture, and gain employee support [25]. The Hospital Survey on Patient Safety Culture was developed in 2004 and updated in 2019 (HSOPSC 2.0) and has been widely used in developed and developing countries. AHRQ recommends authentic translations to ensure cross-cultural consistency, making HSOPSC 2.0 reliable globally [26]. Since its release, it has been translated into 35 different languages and administered in 62 countries worldwide [26]. As a well-validated and widely recognized instrument, we used this tool to thoroughly assess nurses’ patient safety culture in hospitals. It provides meaningful insights and benchmarking opportunities relevant to nursing practice [27].

Research on patient safety culture in Bangladesh remains limited despite global concern [28]. In Bangladesh, studies have reported poor infection control practices, with more than 30% of hospital-acquired infections in some facilities reflecting an inadequate patient safety culture [29]. A national health facility survey found that 21% of inpatients at Upazila Health Complexes, 21% at District Hospitals, and 20.8% at Maternal and Child Health facilities received all prescribed drugs, indicating significant medication safety gaps [30]. Poor safety performance in public hospitals emphasizes the urgent need to strengthen patient safety culture, reduce patient harm, and restore quality in healthcare services [28]. Moreover, nurses’ perceptions of patient safety culture in Bangladesh are unknown, highlighting the need for evidence to improve safety practices and guide healthcare policy [17]. Therefore, this study aimed to culturally adapt a valid and reliable Bengali version of HSOPSC 2.0 to assess patient safety culture among nurses working at government tertiary-level medical college hospitals in Bangladesh.

## Methods

**Fig. 1:**
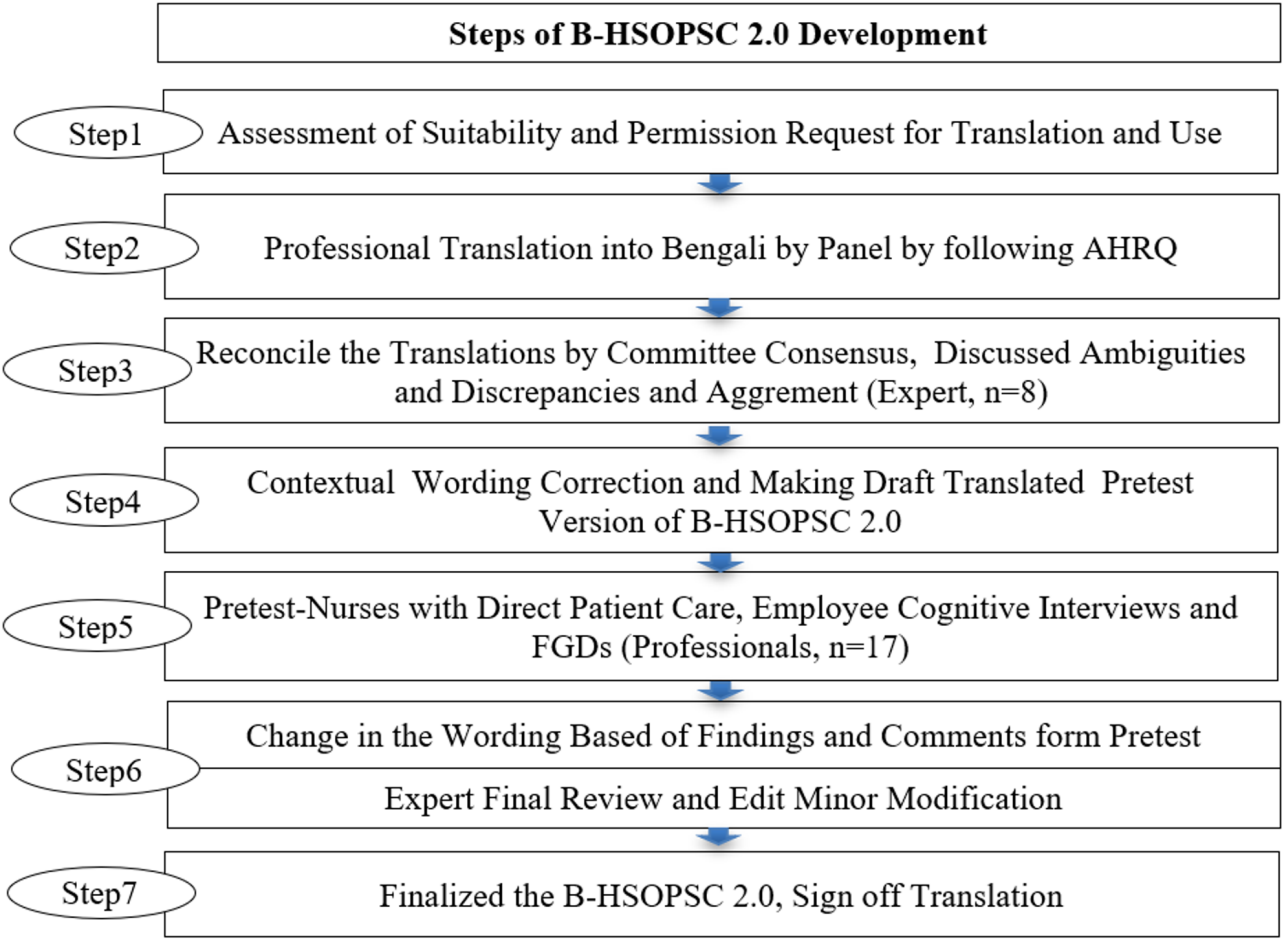
HSOPSC 2.0 Bengali version development procedures

This was a two-step design study comprising translation and cultural adaptations, followed by psychometric evaluation of the Bengali version of Surveys on Patient Safety Culture™ (SOPS^®^) hospital survey 2.0 (B-HSOPSC 2.0). The translation process used a forward-backwards method, and pretesting was conducted in accordance with AHRQ translation guidelines [31]. Further, validity was checked by content and construct validity, and reliability was assessed by internal consistency and test-retest reliability (Fig. 1).

### Setting and sampling

As there are eight administrative divisions in Bangladesh, we conducted our study in eight tertiary-level government medical college hospitals, one per division, to cover the whole country. The study population included all nurses (*N* = 8907) working in different wards of those hospitals. Based on the reference table in the AHRQ guidelines (Appendix 1), our minimum required sample size was 4,575 out of a total of 8,907 nurses from 8 hospitals [32]. In addition to the AHRQ-recommended sample size for survey implementation, the adequacy of the sample size for CFA was considered. The present study included 32 observed variables and an estimated large number of parameters (~ 100). According to commonly accepted recommendations (e.g., 10–20 participants per parameter or ≥ 300 for stable CFA estimation), the obtained sample size (*n* = 4,982) is considered more than sufficient for robust model estimation (Appendix 2). We recruited all nurses who met the inclusion criteria: being nurses with at least 2 years of experience, working for at least 2 months in the current ward, and willing to participate in this study. We excluded nurses who were not involved in direct patient care, those in administrative roles, and those on leave during the data collection period. A total of 7,170 (80.5%) nurses met the inclusion criteria and received questionnaires. Among them, 5,003 nurses completed the survey. Data were checked and cleaned for incomplete and invalid responses. Finally, 4,982 valid responses were retained for analysis, resulting in a valid response rate of 69.5%.

### Study period

Data were collected between April and September 2025.

### HSOPSC 2.0 instruments

The AHRQ HSOPSC 2.0 [33] consists of 34 items. Among all items, 32 items comprised 10 composite dimensions with 5-point Likert scales of agreement (from 1 = strongly disagree to 5 = strongly agree) or frequency (from 1 = never to 5 = always). According to AHRQ guidelines, these scale scores are re-coded as 1–2 = 1, 3 = 2, and 4–5 = 3, used to compute the average percent positive response rate of each dimension. The survey includes 2 single-item outcome measures: frequency of events reported (none = 1; 1 to 2 = 2; 3 to 5 = 3; 6 to 10 = 4; 11 or more = 5) and patient safety rating (from poor = 1, fair = 2, good = 3, very good = 4, and excellent = 5). We re-coded items into 3 levels: poor = 1 (1–2), fair = 2 (3), good/excellent = 3 (4–5). Additionally, we included nine background questions including participants’ age, gender, nursing education, total professional experience, experience in current hospitals, working wards, experience in current wards, in-service training like nursing care, nursing administration, infection prevention and control-related training.

### HSOPSC 2.0 Bengali version (B-HSOPSC 2.0) development procedures

The HSOPSC 2.0 English version was translated into Bengali following the Translation Guidelines established by the AHRQ for the Surveys on Patient Safety Culture™ (SOPS^®^) [31]. The translation procedure was conducted in 7 sequential steps (Fig. 1). First, we obtained written permission from AHRQ to use a translated Bengali version of the English instrument. Then we performed forward and backward translation between English and Bengali, conducted by two bilingual translators who were professors with PhD degrees and experts in translation. Another two bilingual reviewers (experts in patient safety and survey methodology) assessed the translations to ensure clarity, conceptual accuracy, and contextual appropriateness, confirming that the Bengali version faithfully reflected the meaning of the original instrument. The instrument was further reconciled by an expert committee consisting of eight bilingual professionals, including academic faculty with higher education, who possess both clinical and teaching experience; a clinical nurse specialist with expertise in patient safety; and two bilingual translators. All members reviewed the original translation using the master comparison table, which contained forward-backwards-translated comments and reconciled columns. Through detailed discussion and consensus, the committee resolved semantic and cultural discrepancies, ensuring conceptual equivalence with the original instrument. The pre-testing of the instrument was conducted by administrating 17 nurses working at two tertiary medical college hospitals from eight study hospitals. Eleven nurses participated in face-to-face cognitive interviews (retrospective probing) using semi-structured interview guidelines. Another 6 nurses participated in a focus group discussion (FGD). Participants were asked to identify any translated items, response options, or survey instructions that they found confusing or difficult to understand. Notes were taken during interviews and FGDs. Then, the previous translation team (8 experts) again reviewed and incorporated all pretest findings and finalized the Bengali version (B-HSOPSC 2.0).

## Data collection procedures

After obtaining written permission from the respective hospital directors to conduct the research, the Principal Investigator (PI, the first author) convened meetings with the nursing heads, ward in-charges and nurses in the respective hospital conference rooms. The PI explained the study objectives, ethical considerations, and inclusion/ exclusion criteria as well as the role of the participants. Consent was obtained from those who met the eligibility criteria, and questionnaires were distributed. The research assistant (RAs) team assisted and monitored data collection procedures in the wards. Completed questionnaires were collected into secure drop boxes placed in the nurses’ duty rooms and in the nursing supervisor’s room. For retest, data were also collected from 424 participants out of 3727 (11.38%) from three study hospitals, two weeks apart from the main survey.

### Ethical consideration

The study was conducted in accordance with the principles of ethical research in the Declaration of Helsinki 2025 [34]. Ethical approval was obtained from the National Research Ethics Committee of the Bangladesh Medical Research Council (Registration No. 64002032025). Written informed consent was obtained from all the participants.

### Data analysis

The data were analyzed using SPSS for Windows version 0.31.0 (IBM Crop Armonk, NY, USA) and JMP Student Edition version 19.1 (SAS Institute Inc., Cary, NC, USA). Missing data were handled by the listwise method. A complete-case analysis was performed as the proportion of missing values was low (< 5%), and no systematic pattern suggesting bias was observed. All negative items were reverse-coded for analysis. Significance was set at *p* < 0.05. Descriptive statistics were used to evaluate participants’ characteristics and each B-HSOPSC 2.0 subscale [35]. Content validity of the instrument was evaluated using the content validity index; reliability was assessed with Cronbach’s alpha coefficient, and test-retest reliability was determined using the interclass correlation coefficient (ICCs). Construct validity was evaluated using confirmatory factor analysis (CFA), and convergent validity was examined using Spearman’s correlation coefficients.

Reliability was measured with Cronbach’s Alpha, ranging from 0.80 to 1.00, and was considered very good and 0.60–0.79 acceptable [36, 37]. Test-retest reliability was evaluated using ICCs (two-way mixed model, absolute agreement), with ICC > 0.75 considered acceptable [38].

CFA was used to test whether observed variables fit a hypothesized factor structure derived from theory or prior research. In CFA, the cut-off value 0.30 for factor loadings is considered significant when the sample size is ≥ 350 [39].

Items loading below 0.30 may need removal to avoid weak constructs [40]. We used SEM and adopted fit criteria from Byrne (1999), specifying that Goodness of Fit Index (GFI) and Comparative Fit Index (CFI) values ≥ 0.80 indicate acceptable model fit [41, 42]. A Root Mean Square Error of Approximation (RMSEA) value < 0.06 and a Standardized Root Mean Square Residual (SRMR) value < 0.08 were considered a good fit [41]. To provide further evidence of construct validity, Spearman’s correlation coefficient was used to examine the relationships among the dimensions [43, 44].

## Results

### Characteristics of the participants

A total of 4,982 registered nurses participated from the 8 hospitals. The mean age and standard deviation (SD) of the nurses were 37.1 (7.8) years, 26.3% aged ≤ 31 years, 48.8% were 32–41 years, and 24.9% were > 41 years old. Most were female (91.1%). More than half held a nursing diploma (59.8%), followed by a bachelor (26.1%) and a master’s degree or higher (14.2%) (Table 1).

**Table 1:** Socio-demographic and professional characteristics of the participants (*n* = 4982)

Variables	Categories	*N*	(%)	95% CI
Age in years (mean, SD)		(37.1, 7.8)		
	≤ 31	1309	26.3	25.1–27.5
	32–41	2433	48.8	47.4–50.2
	> 41	1240	24.9	23.7–26.1
Gender	Male	441	8.9	8.1–9.7
	Female	4541	91.1	90.3–91.9
Nursing education	Diploma in nursing (3 years of education)	2977	59.8	58.4–61.1
	BSc in nursing (4 years of education)	1299	26.1	24.9–27.3
	Master and above	706	14.2	13.2–15.2
Professional experience in years (mean, SD)		(12.16, 7.14)		
	≤ 7	1318	26.5	25.2–27.7
	8–15	2469	49.6	48.2–51.0
	> 15	1195	24.0	22.8–25.2
Job experience in hospital (years) (mean, SD)		8.62, 6.14)		
	≤ 5	1891	38.0	36.6–39.3
	6–10	1977	39.7	38.3–41.1
	> 10	1114	22.4	21.2–23.5
Working ward	Medicine	1149	23.1	21.9–24.3
	Surgery	690	13.8	12.9–14.8
	Emergency	215	4.3	3.8–4.9
	Intensive care unit	449	9.0	8.2–9.8
	Cardiology/CCU	263	5.3	4.7–5.9
	Operation theater	392	7.9	7.1–8.7
	Orthopedics/orthopedic surgery	218	4.4	3.8–5.0
	Gynecology and obstetrics	428	8.6	7.8–9.4
	Pediatric/ neonatology	576	11.6	10.7–12.5
	Urology/nephrology	206	4.1	3.6–4.7
	Oncology/hematology	189	3.8	3.3–4.4
	Neurology/neurosurgery	207	4.2	3.6–4.7
Job experience in ward (years) (mean, SD)		(3.92, 3.29)		
	≤ 2	2097	42.1	40.7–43.5
	3–5	1849	37.1	35.8–38.5
	> 5	1036	20.8	19.7–21.9

SD=Standard deviation; CCU=Critical care unit.

95% confidence intervals were calculated for descriptive purposes

### Content validity

The content validity of the B-HSOPSC 2.0 was checked by a group of experts (*n* = 6), including academic faculty, clinical nurse specialists, and a patient safety researcher. They individually rated the cultural relevance, clarity, and appropriateness of each translated item using a 4-point scale ranging from 1 (not relevant) to 4 (highly relevant). They recorded the values 1 and 2 = 0 and 3 and 4 = 1. The Bengali version obtained excellent content validity, with all items having a content validity index (I-CVI) range of 0.83-1, all dimensions 0.92-1, and the total scale (34 items) S-CVI = 0.98 (range 0.92-1), indicating good to excellent content validity [45, 46] (Supplementary Table 1).

### Construct validity

The Kaiser-Meyer-Olkin (KMO) test was performed to evaluate sampling adequacy for factor analysis, yielding a value of 0.858, indicating strong shared variance, as values ≥ 0.60 are generally considered acceptable. Bartlett’s Test of Sphericity shows sufficient inter-variable correlations (*p* < 0.001), supporting the data suitability of B-HSOPSC 2.0 with 32 items for factor analysis [47].

**Fig. 2:**
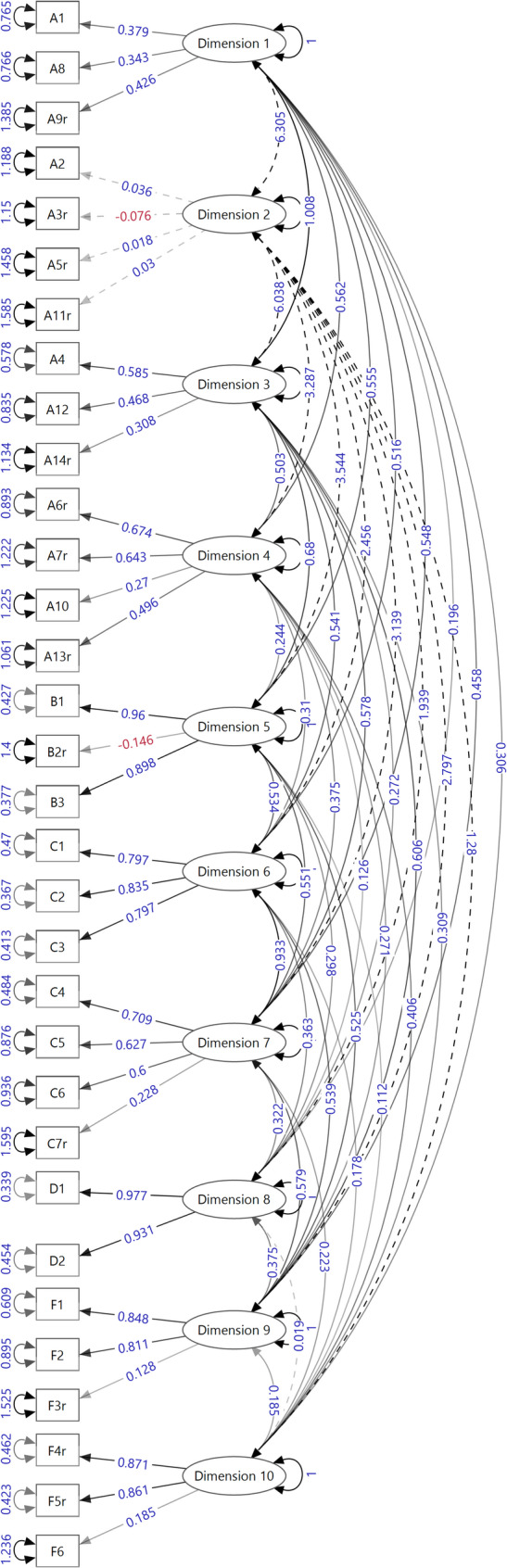
Confirmatory factor analysis of the B-HSOPSC 2.0 scale among nurses (Model 1). CFI = 0.79; NFI = 0.78; TLI = 0.74; GFI = 0.91; RMSEA = 0.06; SRMR = 0.07. D: Dimension, D1: teamwork, D2: Staffing and work pace, D3: organizational learning continuous improvement, D4: response to error, D5: supervisor support for patient safety, D6: communication about error, D7: communication openness, D8: reporting patient safety events, D9: hospital management support for patient safety, D10: handoffs and information exchange

**Fig. 3:**
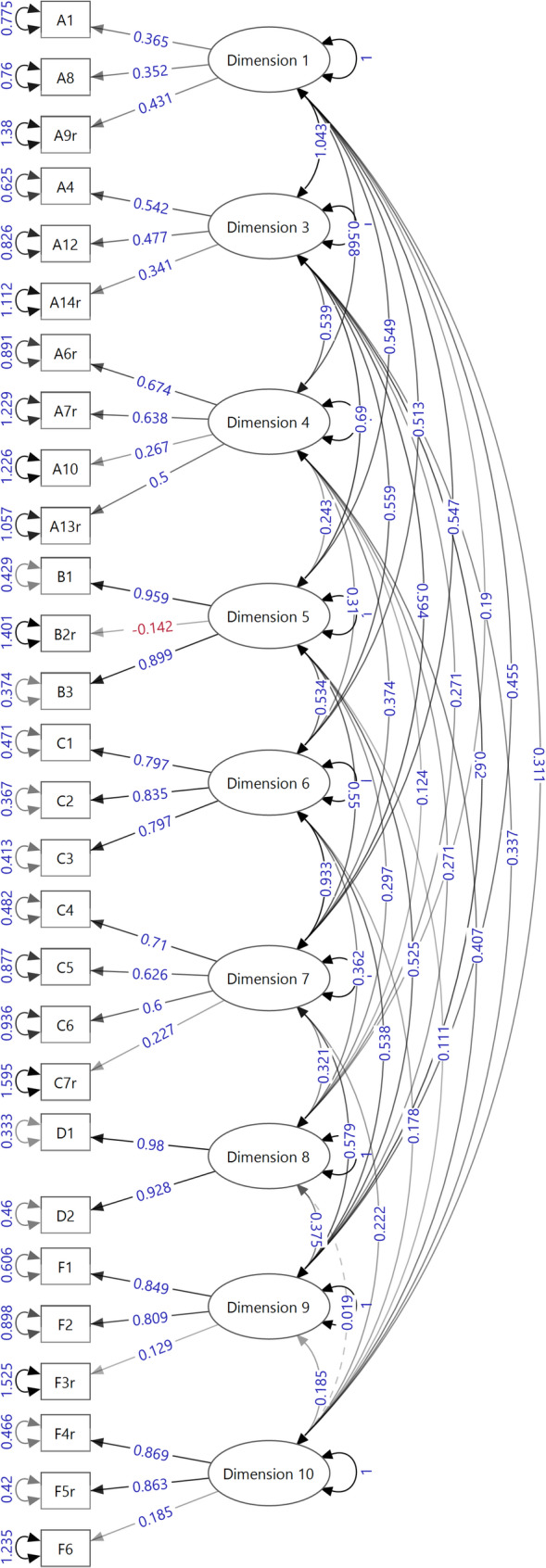
Confirmatory factor analysis of B-HSOPSC 2.0 scale among nurses (Model 2). CFI = 0.83; NFI = 0.82; TLI = 0.79; GFI = 0.92; RMSEA = 0.06; SRMR = 0.06. D: Dimension, D1: teamwork, D3: organizational learning continuous improvement, D4: response to error, D5: supervisor support for patient safety, D6: communication about error, D7: communication openness, D8: reporting patient safety events, D9: hospital management support for patient safety, D10: handoffs and information exchange

CFA was performed to assess construct validity and overall model goodness-of-fit. Two models were developed: one for the original 10-dimensional structure with 32 items (model 1: Fig. 2) and another for the 9-dimensional structure with 28 items (excluding dimension 2 which consists of 4 items) (model 2: Fig. 3). The CFA of the model 1 for B-HSOPSC 2.0 showed mixed fit: CFI (0.79) and Tucker-Lewis Index (TLI) (0.74) indicated suboptimal comparative fit (< 0.90), while GFI (0.90) met acceptable absolute thresholds (≥ 0.90). RMSEA (0.06) and SRMR (0.06) demonstrated excellent fit ≤ 0.06 and ≤ 0.08, respectively. model 1: Fig. 2). In model 1, the factor loading for dimension 2, four items A2, A3r, A5r and A11r factor loading was 0.05, -0.11, 0.003 and 0.04, respectively (< 0.3 is not ideal) low factor loadings (< 0.30) within the “Staffing and Work Pace”, so we removed it and CFA was re-estimate in model 2: Fig. 3. The CFA of model 2 (9 dimensions) for B-HSOPSC 2.0 demonstrated incremental improvement over model 1. Comparative fit indices showed CFI = 0.83 (marginally acceptable, ≥ 0.80) and TLI = 0.79 (below threshold, ≥ 0.90), with NFI = 0.82 indicating marginal fit. Absolute fit was acceptable with GFI = 0.92 (≥ 0.90, improved from Model 1), while RMSEA (0.06, ≤ 0.06) and SRMR (0.06, ≤ 0.08) confirmed excellent fit. These results support the suitability of Model 2 for evaluating patient safety culture among Bangladeshi nurses (Table 2).

**Table 2:** Fit indices for the B-HSOPSC 2.0 (10- and 9-dimension model)

Fit indices	Mode l	Model 2	Threshold	Interpretation
CFI	0.79	0.83	(0.80–0.90)	Model 1 unacceptable Model 2 marginal/acceptable
NFI	0.78	0.82	(≥ 0.80)	Model 1 unacceptable Model 2 marginal
TLI	0.74	0.79	(≥ 0.90)	Poor fit for both models
GFI	0.91	0.92	(≥ 0.90)	Acceptable; Model 2 is slightly better
RMSEA	0.06	0.06	(< 0.06)	Excellent for both
SRMR	0.07	0.06	(< 0.08)	Excellent; Model 2 superior
χ²/df	20.48	20.56	(< 5)	Unacceptable for both

CFI: 0.80–0.90 marginal/acceptable, ≥ 0.90 acceptable, ≥ 0.95 good [47]; NFI: 0.80 marginal, ≥ 0.90 acceptable, ≥ 0.95 good [48]; TLI: Poor fit < 0.90, Marginal/acceptable 0.90–0.95, Good fit > 0.95 [47]; GFI: < 0.90 poor, < 0.95 acceptable, > 0.95 good/excellent [48]; RMSEA: < 0.06 excellent, > 0.06 acceptable, > 0.08 terrible [49]; SRMR: < 0.08 excellent, > 0.08 acceptable, > 0.10 terrible [49]

### Reliability and positive response rate of patient safety culture

Reliability of the Bengali version with 10-dimension scale was checked and an acceptable threshold of Cronbach’s Alpha (α) ≥ 0.70 was obtained for each dimension. The participants’ dimension-specific positive response rates ranged from 22% to 78% compared to U.S. data, ranging from 51% to 82% in 2021. In this study, teamwork achieved the highest rate at 78% (U.S. 82%), whereas reporting patient safety events recorded the lowest rates at 22% (U.S. 74%) and responses were found to be lower compared to the U.S. population (Table 3).

**Table 3:** Reliability statistics and positive response rate of patient safety culture

Subscale (Number of items)	Cronbach’s Alpha		Positive responses (%)	
	This Study	HOSPS 2.0	This Study	Reference (U.S.)
1. Teamwork (3)	0.73	0.76	78	82
2. Staffing and work pace (4)	0.76	0.67	37	58
3. Organizational learning-continuous improvement (3)	0.71	0.76	75	72
4. Response to error (4)	0.72	0.83	54	64
5. Supervisor support for patient safety (3)	0.72	0.77	71	80
6. Communication about the error (3)	0.70	0.89	72	71
7. Communication openness (4)	0.70	0.83	55	51
8. Reporting patient safety events (2)	0.76	0.75	22	74
9. Hospital management support for patient safety (3)	0.71	0.77	62	67
10. Handoffs and exchange information (3)	0.73	0.72	38	64

HOSPSC-Hospital Survey on Patient Safety Culture, U.S.-United States

### Test-retest reliability

We found overall Cronbach’s alpha of the Bengali version with all 34 items was 0.813, indicating acceptable reliability (> 0.70 as acceptable). The test-retest analysis revealed consistent reliability by Cronbach’s alpha (test: α = 0.78; retest: α = 0.78) (data were not shown). The test-retest indicated low single-measure ICCs (0.071, 0.069) (acceptable values 0.6–0.74). However, the average-measure ICCs were 0.717–0.723 (*p* < 0.001), demonstrating acceptable reliability at the group level of the B-HSOPSC 2.0 (Supplementary Table 2) [49].

### Convergent validity and composite reliability

We assessed the convergent validity and composite reliability (CR) of the 10-dimension B-HSOPSC 2.0 and observed that 4 dimensions (5, 6, 8 and 10) met the acceptance threshold. Reporting patient safety events (AVE = 0.91, CR = 0.95) (dimension 8) showed the highest accepted threshold of convergent validity and internal consistency followed by communication about errors (AVE = 0.66, CR = 0.85) (dimension 6), supervisor support (AVE = 0.58, CR = 0.70) (dimension 5) and handoffs and information exchange (AVE = 0.51, CR = 0.71) (dimension 10). The remaining 6 dimensions (1, 2, 3, 4, 7 and 9) did not meet the acceptance thresholds and dimension 3 had the lowest thresholds (Table 4).

**Table 4:** Convergent validity and composite reliability of the ten dimensions of B-HSOPSC 2.0

Dimension with items no	Factor Loading	AVE	CR
1. Teamwork (3)	0.38, 0.34, 0.43	0.15	0.34
2. Staffing and work pace (4)	0.05, -0.11, 0.03, 0.04	0.00	0.00
3. Organizational learning-continuous improvement (3)	0.59, 0.47, 0.31	0.22	0.44
4. Response to error (4)	0.67, 0.64, 0.27, 0.50	0.30	0.61
5. Supervisor support for patient safety (3)	0.96, -0.15, 0.90	0.58	0.70
6. Communication about the error (3)	0.80, 0.83, 0.80	0.66	0.85
7. Communication openness (4)	0.71, 0.63, 0.60, 0.23	0.33	0.64
8. Reporting patient safety events (2)	0.98, 0.93	0.91	0.95
9. Hospital management support for patient safety (3)	0.85, 0.81, 0.13	0.46	0.67
10. Handoffs and exchange information (3)	0.87, 0.86, 0.18	0.51	0.71

AVE: Average Variance Extracted, CR: composite reliability (accepted threshold is AVE ≥ 0.50 and CR ≥ 0.70)

### Correlation among 10-dimension of B-HSOPSC 2.0

Table 5 presents Spearman correlations among 10-dimension of the B-HSOPSC 2.0, ranging from − 0.029 to 0.430. The strongest associations linked communication-related constructs: Communication Openness with Communication About Error (ρ = 0.430, *p* < 0.001) and Supervisor Support (ρ = 0.322, *p* < 0.001). Staffing and Work Pace showed weak or negative correlations (e.g., Teamwork: ρ=-0.029, *p* < 0.05), which confirmed the interconnected structure supporting fit.

**Table 5:** Spearman’s correlation matrix among ten dimensions of the Bengali version of HSOPSC 2.0 (two-tailed)

Dimensions	D1	D2	D3	D4	D5	D6	D7	D8	D9	D10
D1	1.00									
D2	− 0.029^*^	1.00								
D3	0.144^***^	0.01	1.00							
D4	0.120^***^	0.060^***^	0.258^***^	1.00						
D5	0.170^***^	− 0.045^**^	0.237^***^	0.125^**^	1.00					
D6	0.116^***^	-0.01	0.180^***^	0.122^***^	0.345^**^	1.00				
D7	0.129^***^	− 0.034^*^	0.220^***^	0.174^***^	0.322^***^	0.430^***^	1.00			
D8	0.098^***^	-0.01	0.113^***^	0.099^***^	0.192^***^	0.228^***^	0.158^***^	1.00		
D9	0.087^***^	0.02	0.215^***^	0.115^***^	0.239^***^	0.231^***^	0.208^***^	0.202^**^	1.00	
D10	0.089^***^	0.080^***^	0.221^***^	0.151^**^	0.038^**^	0.079^***^	0.141^***^	0.02	0.175^***^	1.00

D: dimension, D1: teamwork, D2: staffing and work pace, D3: organizational learning continuous improvement, D4: response to error, D5: supervisor support for patient safety, D6: communication about error, D7: communication openness, D8: reporting patient safety events, D9: hospital management support for patient safety, D10: handoffs and information exchange

^*^Correlation is significant at the 0.05 level (2-tailed)

^**^Correlation is significant at the 0.001 level (2-tailed)

^***^Correlation is significant at the 0.0001 level (2-tailed)

## Discussion

To the best of our knowledge, this is the first study to conduct a cross-cultural adaptation and psychometric evaluation of the Bengali version of the HSOPSC 2.0 among nurses working at tertiary-level hospitals in Bangladesh, following AHRQ guidelines. The model demonstrated mixed and marginal fit, with acceptable absolute fit indices but suboptimal comparative fit indices. Therefore, this B-HSOPSC 2.0 instrument requires further adjustment and validation. The study included nurses working in hospitals, who represent the largest professional group in the health sector and serve as the first point of contact in patient care. Other studies also evaluated this scale involving nurses with a large sample size in hospital settings [50, 51].

### Translation and cultural adaptation

A rigorous methodological process was undertaken for the translation and validation of B-HSOPSC 2.0. During cultural adaptation, the terms “manager” and “clinical leader” were removed as those terms did not align with the local nursing hierarchy, where senior staff nurses directly report to the nursing superintendent via the nursing supervisor. Similarly, “units” was replaced by “wards,” the standard terminology for functional divisions familiar to the Bangladesh health system [52]. For the item A5r (“on temporary, float, or PRN staff”), a semantic adjustment was made to reflect local practice, where nurses are often reassigned between wards due to a shortage of staff and huge workloads. Therefore, in the final version, it was changed to “on-demand nurses from other wards” and considered contextually more appropriate for the Bangladeshi healthcare context [53]. A similar pattern was seen across both HICs and LMICs, where HSOPSC items were adjusted to reflect better staffing realities and organizational structures, rather than U.S.-based systems [54, 55].

### Validity

The 10-dimension of B-HSOPSC 2.0 demonstrated an excellent level of content validity for items (I-CVI 0.83 to 1.0) and scale (S-CVI 0.98). These findings reflect adaptation relevance, where experts strongly agree with the significance of all dimensions for addressing workforce shortage, suboptimal working conditions and emerging safety culture initiatives, yielding stronger consensus than other high-and middle-income countries (I-CVI-0.73 to 1.0) [56–60]. The CFA results were weak and inconsistent for both models (models 1 and 2). The 10-dimension (model 1) structure of the B-HSOPSC 2.0 has a marginally acceptable but suboptimal model fit. In model 1, we observed dimension 2 had low factor loadings (< 0.30), which is considered unacceptable. Additionally, items in dimensions 4 (A10; when staff make errors, this unit focuses on learning rather than blaming individuals), 5 (B2r; my supervisor wants us to work faster during busy times, even if it means taking shortcut), 7 (C7r; in this unit, staff are afraid to ask questions when something does not seem right), 9 (F3r; hospital management seems interested in patient safety only after an adverse event happens), and 10 (F6; during shift changes, there is adequate time to exchange all key patient care information) had low factor loading. These findings reflect the critical nurses’ shortage, where Bangladesh has 0.6 nurses and midwives per 1,000 population compared to the WHO-recommended 4.45 per 1,000 [61]. Moreover, nurses manage 50–70% more patients compared to the hospital bed capacity [62]. Low factor loadings might be influenced by the hierarchical blame culture and heavy workloads in public hospitals. A blame-oriented approach prioritizes individual accountability over learning (A10) [63], while understaffing normalizes supervisory pressure for work speed (B2r). Hierarchical structures discourage questioning, reduce the openness of communication (C7r), centralized decision-making yields reactive management (F3r) [64], and overcrowding and heavy workloads limit handoffs and transitions (F6) [65].

Therefore, we conducted model 2 with 9 dimensions by removing dimension 2 and found a relatively better model fit, with most of the fit indices showing improvement, although several remained below recommended thresholds. CFI and NFI increased from unacceptable to marginal/acceptable (0.79, 0.78 and 0.83, 0.82, respectively) in model 2 from model 1. However, the TLI was found to be below the acceptable threshold (< 0.80), and the χ²/df ratios were unacceptably high (≥ 5) for both models. Although model 2 with 9 dimensions showed a better fit, dimension 2 remains important because it helps identify systemic problems in public hospitals that directly threaten patient safety. There might be some measurement limitations that can lead to weak and inconsistent results in CFA. As nurses fill up the questionnaire themselves, question items might be interpreted differently, leading to inconsistencies in responses. They answered in a way they thought was favorable rather than how they truly felt, affecting the accuracy of their responses. Moreover, we conducted this study throughout the country, question items that were not used to with local cultural context/dialect, lead to misunderstandings and affect the validity of the results. We used Likert scale which might limit the response of full spectrum of feelings and experiences. Measurement invariance across different groups (e.g. by age, gender, years of experience) might also affect the CFA results. As our data were not normally distributed, it might also affect the results and lead to weak and inconsistent findings. The studies conducted on resource shortages in LMICs versus stable structures in higher-income countries (HICs) showed mixed CFA fits and model modifications across countries reflect context-specific healthcare realities [57, 58, 66, 67]. Both HICs and LMICs adaptations removed several items and composites, reflecting context-specific differences of psychometric properties in the scale structure. The AVE results showed that convergent validity was substantially weak and only 4 out of 10 dimensions that met the AVE ≥ 0.50 threshold, indicating that several constructs may not be adequately captured by their items, consistent with findings reported in other studies [66, 68].

The observed correlation pattern provides evidence of construct validity for the Bengali version. Communication- and leadership-related dimensions demonstrated positive inter-relationships consistent with shared day-to-day safety practices, whereas Staffing and work pace showed weak correlations, likely reflecting the contextual normalization of workload pressures in Bangladesh’s resource-constrained and overcrowded health system rather than measurement failure. Incongruent findings are reported in HICs, where flexible resources, temporary staffing, and process optimization yield balanced correlations among 10-dimension without major outliers [58].

Therefore, low factor loadings and AVE values below the accepted threshold in 10-dimension (model 1) and removal of dimension 2 (model 2) suggest the adapted instrument does not fully achieve the model structure. The current version of the structure requires further refinement with diverse healthcare professionals before it can be considered fully established. Despite being below the recommended thresholds of dimension 2, we retained it to preserve the integrity of the composite measure and ensure cross-study comparability, as this captures key elements of patient safety culture that are particularly relevant in the Bangladeshi healthcare context and internal consistency (alpha ≥ 0.70).

### Reliability

We found the 10-dimension had acceptable reliability ranging from (Cronbach’s α = 0.70–0.76) and good test–retest reliability at the group level (average-measure ICC = 0.717–0.723) However, single-measure ICC values were extremely low (≈ 0.07), indicating poor reliability for individual-level scores and suggesting that the instrument is more appropriate for group- or unit-level assessment (e.g., hospital, ward, or department) than for evaluating individual nurses’ safety perceptions. These findings reflect that dimensions and items of B-HSOPSC 2.0 were suitable for evaluating patient safety culture at the hospital level by nurses in Bangladesh. In contrast, other studies conducted in HICs and LMICs observed diverse reliability (Cronbach’s α = 0.67–0.89), which might be for flexible staffing and stable safety initiatives, with stronger communication and teamwork with differences in resources related dimensions D2, D3, D4 and D10 [49, 57, 69, 70]. Additionally, four dimensions met the CR threshold (≥ 0.70), driven by strong loadings on modifiable, behaviour-based safety practices, while resource-dependent dimensions showed lower CRs, consistent with LMIC constraints. Therefore, based on our study findings, Cronbach’s alpha, ICCs and CR indicate the overall reliability of B-HSOPSC 2.0 among hospital nurses in Bangladesh.

### Positive response rate of patient safety culture

We compared the 10-dimension-wise positive responses with the U.S. HSOPSC 2.0 data as a reference and found Bangladeshi nurses scored higher on dimensions 1, 3, 6 and 7 compared to the U.S. population. We found the highest responses for dimension 1 (teamwork) and the lowest for dimension 8 (reporting patient safety events). Therefore, special attention needs to be given to improve the reporting system of patient safety events through teamwork among nurses and other healthcare professionals. Similar findings from LMIC studies reported low scores for Staffing and Work Pace (Dimension 2), consistent with our results and notably lower than those reported in U.S. (HIC) studies [49, 67, 71].

### Strengths

This is the first validated and reliable Bengali version of the HSOPSC 2.0, addressing a critical gap in assessing patient safety culture among hospital nurses in Bangladesh. The culturally adapted tool enhances accuracy in measuring safety perceptions, supports cross-national comparability, and offers evidence for developing locally relevant instruments while guiding quality improvement efforts.

### Limitations

This study employed purposive sampling limited to nurses from eight Bangladeshi government medical college hospitals, excluding other professional medical staff members (e.g., physicians, laboratory and supporting staff), which may introduce social desirability bias due to reliance on self-reports. We collected data only from nurses, which limits the applicability of this result to other healthcare professionals. Psychometric evaluation also revealed several limitations: model fit indices (CFI, TLI) were below recommended thresholds, and the higher χ²/df ratio indicated model misfit. Convergent validity was limited, and test–retest reliability, as measured by ICC values, was extremely low at the single-level, warranting cautious interpretation. Future research needs diverse and more representative samples across different levels of hospitals and professional groups, along with concurrent validity testing against established gold-standard scales to strengthen the evidence for the Bengali HSOPSC 2.0.

### Future research

Future studies should aim to achieve higher adherence rates and include multi-professional samples to strengthen the robustness of CFA results amid mixed fit. Additionally, this study highlights priority areas that include enhancing work environments and reporting systems to advance patient safety culture.

## Conclusions

This study translated the original English HSOPSC 2.0 scale into Bengali and validated its suitability for use among nurses in Bangladeshi hospitals. The Bengali version showed acceptable content and construct validity. The 10-dimension, 32-item Bengali version of HSOPSC 2.0 found a weak and inconsistent model fit; however, the 9-dimension (without dimension 2) model with 28 items was found to be a better fit than 10-dimension for nurses working at the hospitals in Bangladesh. All 32 items and their respective subscales demonstrated acceptable reliability, with group-level test–retest reliability. While important psychometric limitations remain unresolved, the study offers valuable initial evidence for a culturally adapted instrument in this under-researched context. Among the factor structure (10 vs. 9 dimensions), B-HSOPSC 2.0 with 9-dimension structure can be considered as a preliminary and context-specific tool to evaluate patient safety culture for this group. As dimension 2 (Staffing and work pace) is an essential component for the development of patient safety culture, especially in resource-poor settings, policymakers should take proper initiatives to improve hospital staffing and work pace systems. In the absence of a culturally adapted, reliable Bengali HSOPSC 2.0, this tool fills an important gap by providing a standardized measure to lead hospital safety assessment and policy decisions. However, its psychometric properties among physicians and other allied health professionals remain untested. Further research, including diverse and more representative samples across different levels of hospitals and professional groups, will help further refine and strengthen the psychometric evidence for the Bengali version of HSOPSC 2.0 in the Bangladeshi context.

## Supplementary Information

Below is the link to the electronic supplementary material.

Supplementary Material 1

Supplementary Material 2

Supplementary Material 3

## Data Availability

The datasets generated and/or analyzed during the current study are not publicly available due to the confidentiality of the participants. Data are available from the corresponding author upon reasonable request.

## Abbreviations

HSOPSC: Hospital Survey on Patient Safety Culture
PSC: Patient safety culture
PS: Patient safety
CFA: Confirmatory factor analysis
CFI: Comparative fit index
GFI: Goodness of fit index
RMSEA: Root mean square error of approximation
SRMR: Standardized root mean square residual
