# Supplementary material for: Cross-cultural adaptation and validation of hospital survey on patient safety culture in Bengali version (B-HSOPSC 2.0) among nurses in Bangladesh: a cross-sectional study
Source: BMC Nurs. 2026 May 29;25:679. doi: 10.1186/s12912-026-04817-3 (PMC13435589; doi:10.1186/s12912-026-04817-3)
Supplement: Supplementary file 1 [file 12912_2026_4817_MOESM1_ESM.docx]

**Supplementary Table 1** Content Validity Index for each item (I-CVI) and Scale (S-CVI) for each dimension

| **Items (32)** | **Description** | **I-CVI** |
| --- | --- | --- |
| **Dimension 1: Teamwork (S-CVI=1.00**) | | |
| A1 | In this unit, we work together as an effective team. | 1.00 |
| A8 | During busy times, staff in this unit help each other. | 1.00 |
| A9r | There is a problem with disrespectful behaviour by those working in this unit. | 1.00 |
| **Dimension 2: Staffing and work pace (S-CVI=0.96**) | | |
| A2 | In this unit, we have enough staff to handle the workload. | 1.00 |
| A3r | Staff in this unit work longer hours than is best for patient care. | 0.83 |
| A5r | This unit relies too much on temporary, float, or PRN staff. | 1.00 |
| A11r | The work pace in this unit is so rushed that it negatively affects patient safety. | 1.00 |
| **Dimension 3: Organizational learning-continuous improvement (S-CVI=1.00)** | | |
| A4 | This unit regularly reviews work processes to determine if changes are needed to improve patient safety. | 1.00 |
| A12 | In this unit, changes to improve patient safety are evaluated to see how well they worked. | 1.00 |
| A14r | This unit lets the same patient safety problems keep happening. | 1.00 |
| **Dimension 4: Response to error (S-CVI=1.00)** | | |
| A6r | In this unit, staff feel like their mistakes are held against them. | 1.00 |
| A7r | When an event is reported in this unit, it feels like the person is being written up, not the problem. | 1.00 |
| A10 | When staff make errors, this unit focuses on learning rather than blaming individuals. | 1.00 |
| A13r | In this unit, there is a lack of support for staff involved in patient safety errors. | 1.00 |
| **Dimension 5: Supervisor support for patient safety (S-CVI=0.94)** | | |
| B1 | My supervisor seriously considers staff suggestions for improving patient safety. | 1.00 |
| B2r | My supervisor wants us to work faster during busy times, even if it means taking shortcuts. | 0.83 |
| B3 | My supervisor takes action to address patient safety concerns that are brought to their attention. | 1.00 |
| **Dimension 6: Communication about error (S-CVI=1.00)** | | |
| C1 | We are informed about errors that happen in this unit. | 1.00 |
| C2 | When errors happen in this unit, we discuss ways to prevent them from happening again. | 1.00 |
| C3 | In this unit, we are informed about changes that are made based on event reports. | 1.00 |
| **Dimension 7: Communication openness (S-CVI=0.92**) | | |
| C4 | In this unit, staff speak up if they see something that may negatively affect patient care. | 1.00 |
| C5 | When staff in this unit see someone with more authority doing something unsafe for patients, they speak up. | 0.83 |
| C6 | When staff in this unit speak up, those with more authority are open to their patient safety concerns. | 0.83 |
| C7r | In this unit, staff are afraid to ask questions when something does not seem right. | 1.00 |
| **Dimension 8: Reporting patient safety events (S-CVI=1.00)** | | |
| D1 | When a mistake is caught and corrected before reaching the patient, how often is this reported? | 1.00 |
| D2 | When a mistake reaches the patient and could have harmed the patient, but did not, how often is this reported? | 1.00 |
| **Dimension 9: Hospital management support for patient safety (S-CVI=0.94)** | | |
| F1 | The actions of hospital management show that patient safety is a top priority. | 1.00 |
| F2 | Hospital management provides adequate resources to improve patient safety. | 1.00 |
| F3r | Hospital management seems interested in patient safety only after an adverse event happens. | 0.83 |
| **Dimension 10: Handoffs and information exchange (S-CVI=1.00)** | | |
| F4r | When transferring patients from one unit to another, important information is often left out. | 1.00 |
| F5r | During shift changes, important patient care information is often left out. | 1.00 |
| F6 | During shift changes, there is adequate time to exchange all key patient care information. | 1.00 |
| **Items (2)** |  |  |
| D3 | In the last 12 months, how many patient safety events have you reported? | 1.00 |
| E1 | How would you rate your unit/work area for patient safety? | 1.00 |
| **S-CVI-A** |  | **0.98** |

r: negatively worded items, S-CVI-A: Average of content validity index of scale with all items

**Supplementary Table 2** Test-retest reliability and intraclass correlation coefficients (ICCs) for B-HSOPSCC 2.0

| **Intraclass correlation b** | | | **95% Confidence interval** | | **F Test** | **Sig** |
| --- | --- | --- | --- | --- | --- | --- |
|  |  |  | **Lower bound** | **Upper bound** | **Value** |  |
| **Test** | Single measures | .071a | 0.06 | 0.09 | 4.61 | <.001 |
|  | Average measures | .723c | 0.68 | 0.76 | 4.61 | <.001 |
| **Retest** | Single measures | .069a | 0.06 | 0.09 | 4.52 | <.001 |
|  | Average measures | .717c | 0.67 | 0.76 | 4.52 | <.001 |

Two-way mixed effects model where people’s effects are random and measures effects are fixed. a

The estimator is the same, whether the interaction effect is present or not. b

Type A intraclass correlation coefficients using an absolute agreement definition. c

This estimate is computed assuming the interaction effect is absent because it is not estimable otherwise.
