# Supplementary material for: Cross-cultural adaptation and validation of hospital survey on patient safety culture in Bengali version (B-HSOPSC 2.0) among nurses in Bangladesh: a cross-sectional study
Source: BMC Nurs. 2026 May 29;25:679. doi: 10.1186/s12912-026-04817-3 (PMC13435589; doi:10.1186/s12912-026-04817-3)
Supplement: Supplementary file 2 [file 12912_2026_4817_MOESM2_ESM.docx]

**Appendix 1**

**Sample size calculation**

We calculated our required sample size by following the AHRQ guidelines:

AHRQ guidelines reference table [31]

| **Number of Providers and Staff** | **Minimum Sample Size*** | **Expected Number of Responses (Assuming a 50% Response Rate)** |
| --- | --- | --- |
| 500 (or fewer) | 500 – a census of all providers and staff | 250 (or fewer) |
| 501–699 | 500 | 250 |
| 700–1,299 | 600 | 300 |
| 1,300–3,999 | 700 | 350 |
| 4,000 or more | 750 | 375 |

*The sample size is based on three assumptions: simple random or systematic random sampling, a response rate of 50 percent, and a confidence interval of +/- 5 percent.

**Enrolment of samples from eight different hospitals**

| **Name of the hospitals** | **Total number of nurses in each hospital** | **Minimum sample required as per AHRQ guidelines** | **Actual sample collected by hospitals** |
| --- | --- | --- | --- |
| Dhaka Medical College Hospital | 2525 | 700 | 1119 |
| Chattrogram Medical College Hospital | 1166 | 600 | 623 |
| Rajshahi Medical College Hospital | 1178 | 600 | 816 |
| Mymensingh Medical College Hospital | 1063 | 600 | 630 |
| Khulna Medical College Hospital | 475 | 475 | 354 |
| Sher-e-Bangla Medical College Hospital | 929 | 500 | 529 |
| Rangpur Medical College and Hospital | 992 | 600 | 503 |
| Sylhet MAG Osmani Medical College Hospital | 579 | 500 | 429 |
| **Total** | **8,907** | **4,575** | **5,003** |
