# Supplementary material for: Cross-cultural adaptation and validation of hospital survey on patient safety culture in Bengali version (B-HSOPSC 2.0) among nurses in Bangladesh: a cross-sectional study
Source: BMC Nurs. 2026 May 29;25:679. doi: 10.1186/s12912-026-04817-3 (PMC13435589; doi:10.1186/s12912-026-04817-3)
Supplement: Supplementary file 3 [file 12912_2026_4817_MOESM3_ESM.docx]

**Appendix 2**

**Estimated sample for confirmatory Factor Analysis**

| **Item** | **Value** |
| --- | --- |
| Number of observed variables | 32 |
| Estimated parameters | ~100 (approx.) |
| Recommended sample size (10–20 per parameter) | 1000–2000 |
| Actual sample size | 4982 |
| Adequacy | Sufficient |
